# Supplementary material for: An analysis modality for vascular structures combining tissue-clearing technology and topological data analysis
Source: Nat Commun. 2022 Sep 12;13:5239. doi: 10.1038/s41467-022-32848-2 (PMC9468184; doi:10.1038/s41467-022-32848-2)
Supplement: Supplementary file 1 — Supplementary Information [file 41467_2022_32848_MOESM1_ESM.pdf]

Supplementary Table 1. Comparison of NHPP and TH with existing methods

<table>
<tr><th>Methods</th><th>Structural information obtained</th><th>Missing structural information</th><th colspan="2">Other limitations</th></tr>
<tr><td>TubeMap</td><td>Branching/end points, length and radius of branches</td><td>Spatial information</td><td></td><td rowspan="3">Quality of original images and classification process may partially affect results</td></tr>
<tr><td>NHPP</td><td>Signal densities and their directionalities (X-, Y-, Z-)</td><td rowspan="2">Structural parameters such as branching points and radius of branches</td><td rowspan="2">High computational cost</td></tr>
<tr><td>TDA</td><td>Overall structure from the "shape" of the data, while not depending on the choice of metrics and providing stability against noise</td></tr>
</table>

Supplementary Table 2. Information of computer specification

Purpose	RAM	Time
Ilastik	256 GB	Lung: 1-3 h/sample
Python	256 GB	Depending on the code<br>e.g., file conversion: < 10-20 min/sample
Imaris software	For creating multi-color files or movies: 128 or 256 GB<br>For viewing: 16 GB	Creating multi-color file: 10-20 min/sample
NHPP	128 GB	1 d/sample
Persistent homology	128 GB	10-20 min/sample

Supplementary Table 3. Time of CUBIC procedures

<table>
<tr><th>mouse body/organ</th><th>CUBIC-L</th><th>staining</th><th>decalcification</th><th>CUBIC-R</th></tr>
<tr><td>whole mouse</td><td>5-7 d</td><td>5-7 d</td><td rowspan="10">(-)</td><td rowspan="12">1-2 d</td></tr>
<tr><td>lung</td><td rowspan="5">1-3 d</td><td rowspan="10">3-4 d</td></tr>
<tr><td>stomach</td></tr>
<tr><td>intestine</td></tr>
<tr><td>pancreas</td></tr>
<tr><td>eye</td></tr>
<tr><td>brain</td><td rowspan="4">2-4 d</td></tr>
<tr><td>heart</td></tr>
<tr><td>kidney</td></tr>
<tr><td>spleen</td></tr>
<tr><td>bone</td><td>1-3 d</td><td>1 week</td></tr>
<tr><td>liver</td><td>5-7 d</td><td>5-7 d</td><td>(-)</td></tr>
</table>

Supplementary Table 4. Antibody information

<table>
<tr><th>antibody</th><th>Supplier</th><th>catalog#</th><th>dilution</th></tr>
<tr><td>anti-GFP conjugated with Alexa Fluor 594</td><td rowspan="3">ThermoFisher Scientific</td><td>A21312</td><td rowspan="2">1:100-1:200</td></tr>
<tr><td>anti-GFP conjugated with Alexa Fluor 647</td><td>A31852</td></tr>
<tr><td>anti-a-SMA-FITC</td><td>F3777</td><td>1:200-1:400</td></tr>
<tr><td>anti-VEGFR3</td><td>R&D systems</td><td>AF743</td><td>1:100-1:200</td></tr>
<tr><td>Alexa Fluor 647 (2nd antibody)</td><td>ThermoFisher Scientific</td><td>A21447</td><td>1:100-1:200</td></tr>
</table>

Supplementary Figure 1

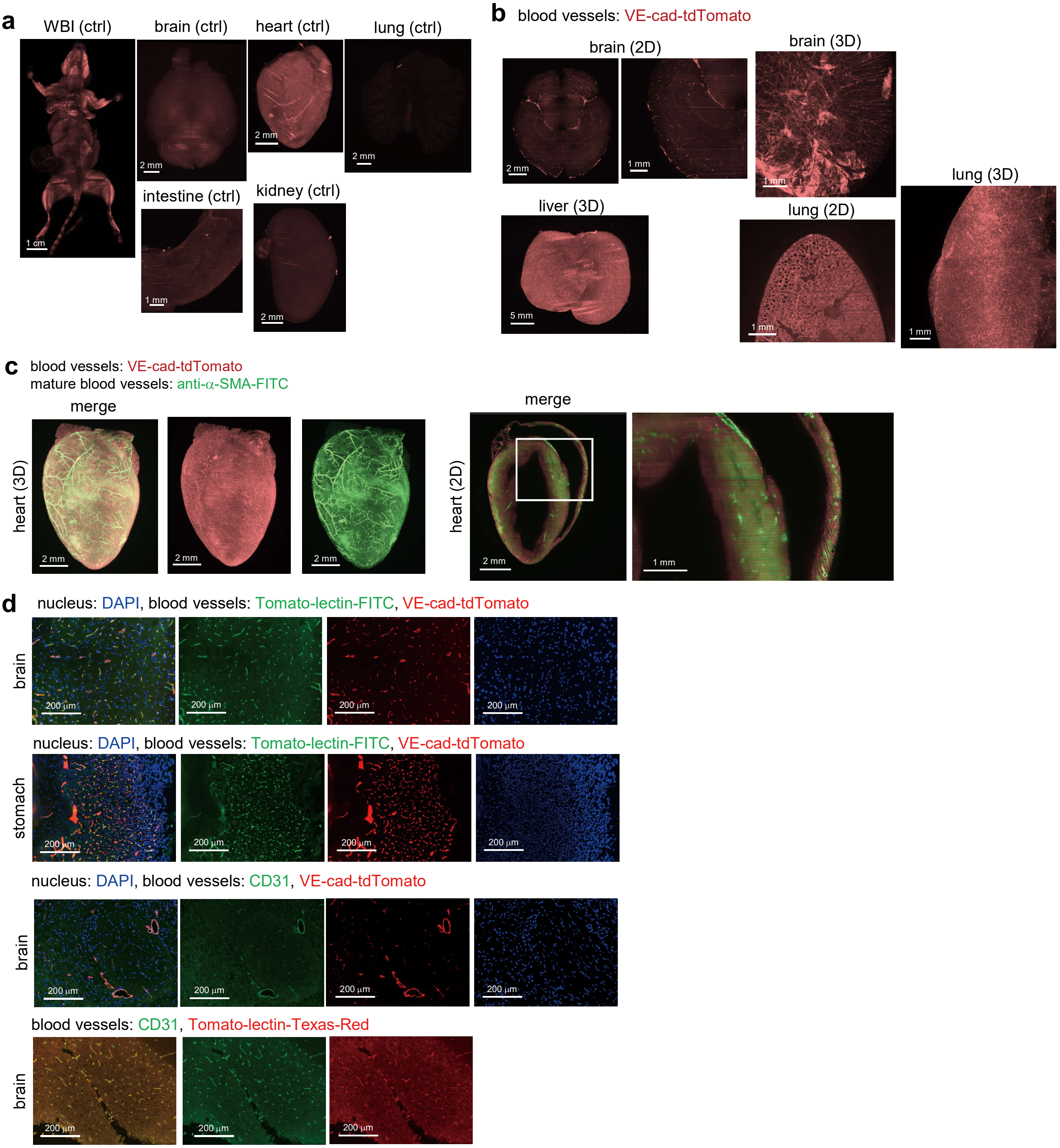

Supplementary Fig. 1 Visualization of blood vessels by CUBIC procedures.

a The 3D images of the whole mouse and various organs in the control mice (ctrl). Normal mice (female, 4 months) were subjected to CUBIC procedures. The 3D images of the whole body, brain, heart, intestine, kidney, and lung are shown.

b The 3D and 2D (XY) images of VE-cad-tdTomato mice (2-4 months). The images of the brain, liver, and lung are shown.

c Simultaneous visualization of α-SMA⁺ and VE-cad⁺ blood vessels in the heart. VE-cad-tdTomato mice (10-11 months) were sacrificed, and samples were subjected to CUBIC procedures. Samples were stained with anti-α-SMA-FITC antibody. The 3D whole-heart images and 2D (XY) images are shown. The enlarged 2D images of white insets are shown in the right panels (Z = 10 μm step, digital zoom; 2.0).

d Expression of blood vasculature markers determined by 2D immunohistochemistry (IHC). Before sacrificing, tomato-lectin conjugated with FITC was injected in VE-cad-tdTomato mice (2-8 months), to which Tx were injected. The brain and stomach were excised. Frozen sections were stained with anti-CD31 antibody and encapsulated with a mounting agent containing DAPI. Tomato-lection conjugated with Texas-Red was injected into normal mice (3-4 months) before sacrifice, and samples were frozen. Frozen sections were stained with anti-CD31 antibody. Representative images (brain and stomach) are shown.

Supplementary Figure 2

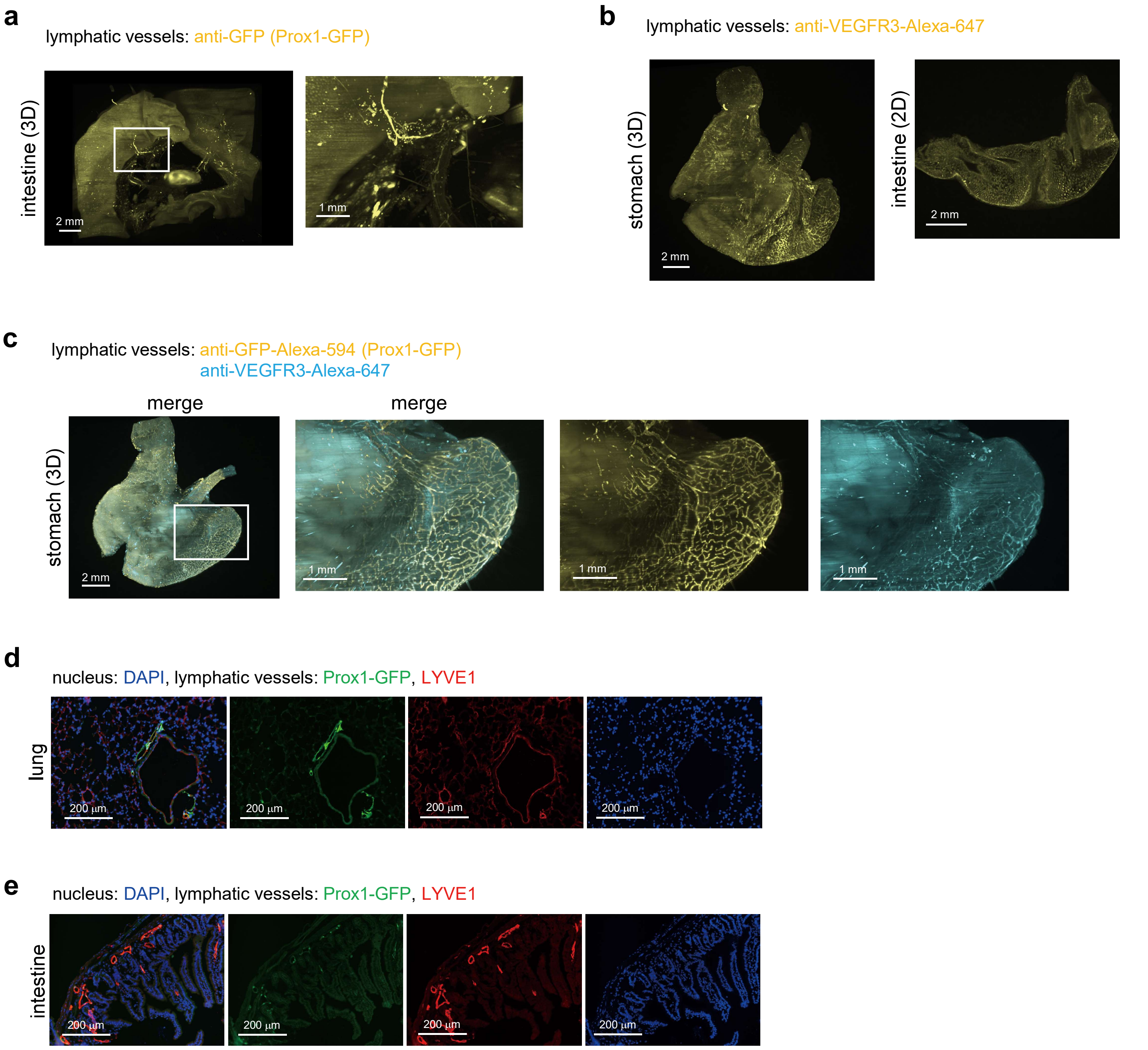

Supplementary Fig. 2 Visualization of lymphatic vessels by CUBIC procedures.

a The 3D images of the intestine and mesentery in Prox1-GFP mice. The intestine with mesentery from Prox1-GFP mice (2-3 months) was embedded into 2% gel before RI adjustment (Z = 10 μm step, digital zoom: 2.0).

b The 3D whole-organ images of the lymphatic vessels with VEGFR3 staining. Samples were stained with anti-VEGFR3 antibody. The 3D image of the stomach and the 2D image of the intestine are shown (Z = 10 μm step, digital zoom; stomach: 1.25, intestine: 2.0).

c The signal confirmation of lymphatic vessels. The 3D images of the organs stained with anti-VEGFR3 antibody are shown. The stomach of Prox1-GFP mouse (3-4 months) was subjected to CUBIC procedures and stained with anti-VEGFR3 antibody (Z = 10 μm step, digital zoom: 1.6).

d and **e** Expression of lymphatic vascular markers determined by 2D IHC. Prox1-GFP mice (1-2 months) were sacrificed, and samples were frozen with O.C.T. compound. Frozen sections were stained with anti-LYVE1 antibody. Representative images of the lung are shown (**d**). LYVE1 expression in the intestine was also determined by staining with anti-LYVE1 antibody (**e**). Representative data from 2 independent experiments are shown.

Supplementary Figure 3

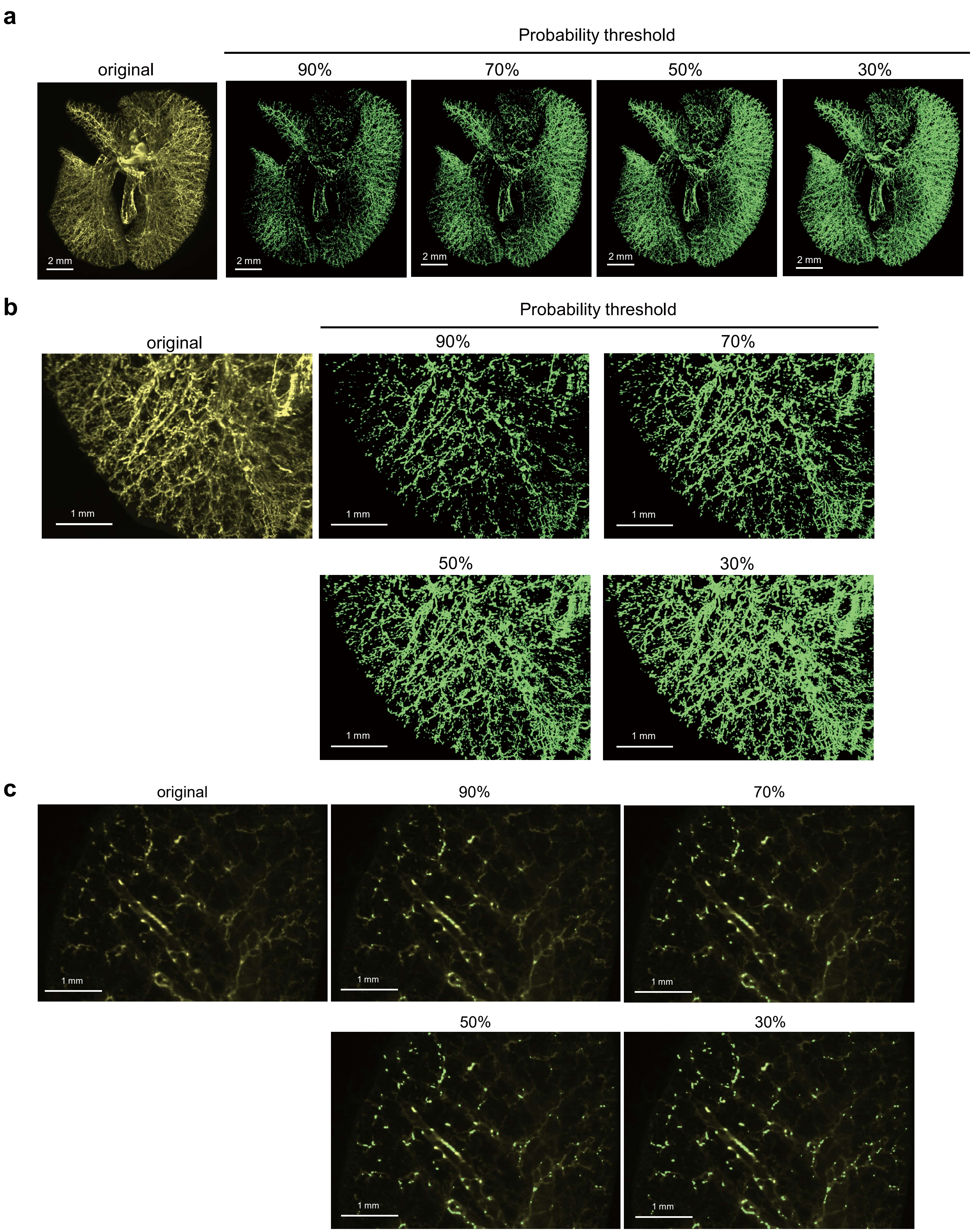

Supplementary Fig. 3 Pixel classification and threshold of their probability.

The 3D whole-lung images (essentially the same with Fig. 3b and originated from control#1 in Fig. 7) from the original and classified signals at each threshold of probability (90%, 70%, 50%, 30%) shown in Figure 4b. The 3D images of whole-lung (**a**), enlarged 3D images (**b**) and 2D images (**c**) are shown. Yellow signals indicate the lymphatic vessels from the original images and green signals indicate the signals classified as lymphatic vessels.

Supplementary Figure 4

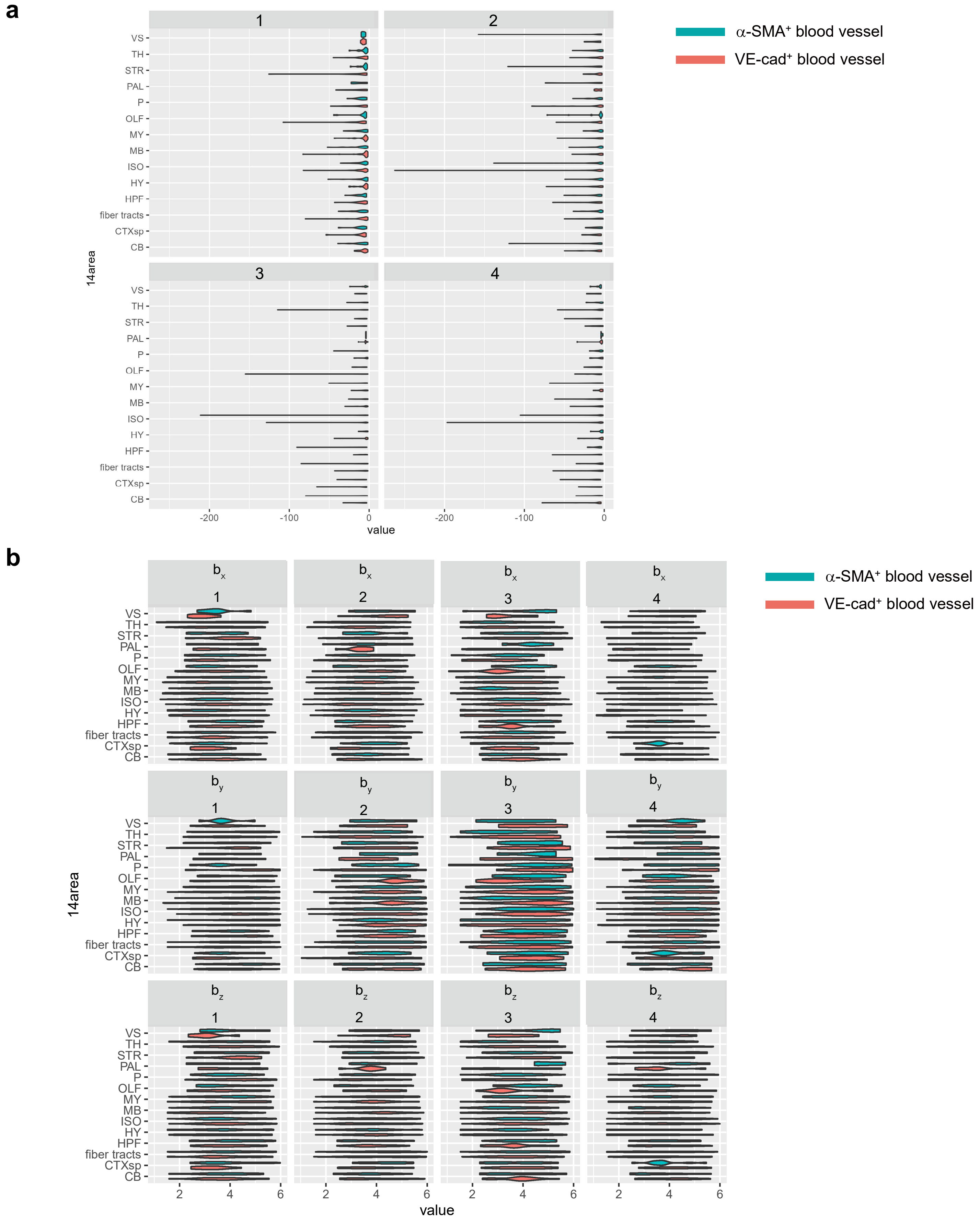

Supplementary Fig. 4 Analysis of brain blood vessels using NHPP.

The classified signals as blood vessels were subjected to the NHPP analysis. The strength (a) of α-SMA⁺ blood vessels or VE-cad⁺ blood vessels are shown in each brain area (**a**). The directionalities (b_x, b_y, b_z) of α-SMA⁺ blood vessels or VE-cad⁺ blood vessels are shown in each brain area (**b**) (14 brain areas: cerebellum (CB), cortical subplate (CTXsp), fiber tracts (fiber), hippocampal formation (HPF), hypothalamus (HY), isocortex (ISO), midbrain (MB), medulla (MY), olfactory areas (OLF), pons (P), pallidum (PAL), striatum (STR), thalamus (TH), and ventricular systems (VS)). Data from four different mouse brains are shown (blue bar: α-SMA⁺ blood vessels, red bar: VE-cad⁺ blood vessels). Source data are provided as a Source Data file.

Supplementary Figure 5

mature blood vessels: anti-α-SMA-FITC
blood vessels: VE-cad-tdTomato

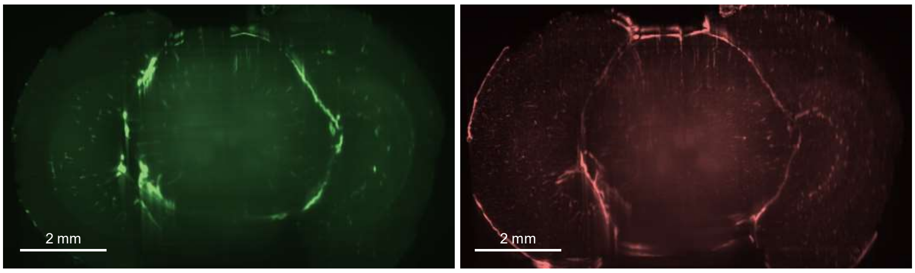

Supplementary Fig. 5 The 2D images of brain blood vessels.

The 2D images of the original images with α-SMA⁺ and VE-cad⁺ signals in the brain. The representative 2D images (coronal view, XZ) are shown.

Supplementary Figure 6

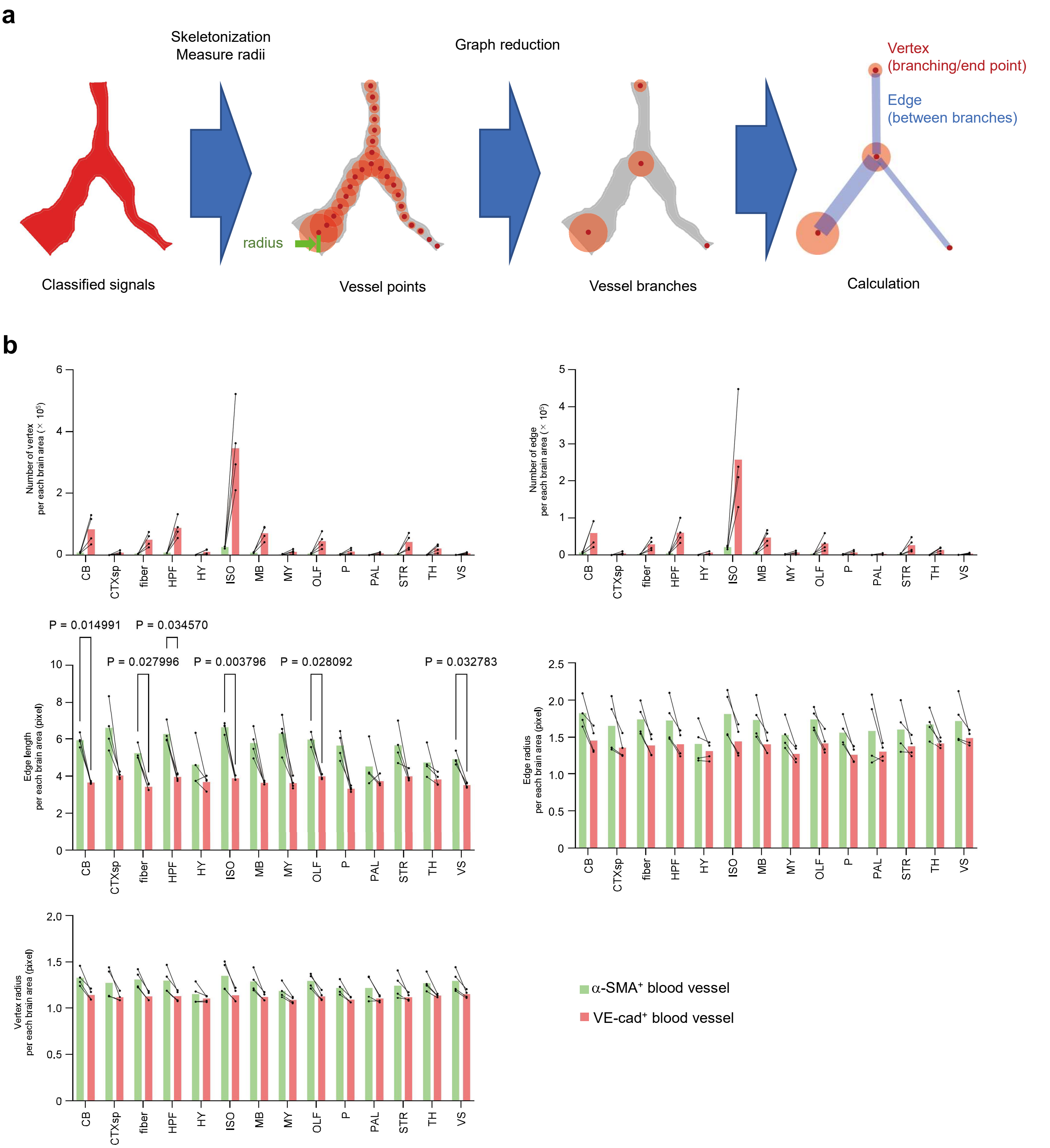

Supplementary Fig. 6 Evaluation of branching points using TubeMap.

a Overview of TubeMap analysis. TubeMap is a platform for quantifying vasculature structures especially in brain [20]. Classified signals as vasculature are replaced by spheres, and vessel branching points and end points are extracted. Vertex (branching points and end points) and edge (between branching and/or end points) are defined. The number of vertex and edge, the length of edge, and the radii of vertex and edge can be calculated.

b The number of vertex and edge, edge length and radii, and vertex radii of α-SMA$^+$ blood vessels and VE-cad$^+$ blood vessels in each brain area are shown (n = 4) (14 brain areas: cerebellum (CB), cortical subplate (CTXsp), fiber tracts (fiber), hippocampal formation (HPF), hypothalamus (HY), isocortex (ISO), midbrain (MB), medulla (MY), olfactory areas (OLF), pons (P), pallidum (PAL), striatum (STR), thalamus (TH), and ventricular systems (VS)). Data from four different mouse brains are shown (green bar: α-SMA$^+$ blood vessels, red bar: VE-cad$^+$ blood vessels). Paired-samples t-test (two-sided) is performed and Bonferroni-Dunn method was used for multiple testing correction. Source data are provided as a Source Data file.

Supplementary Figure 7

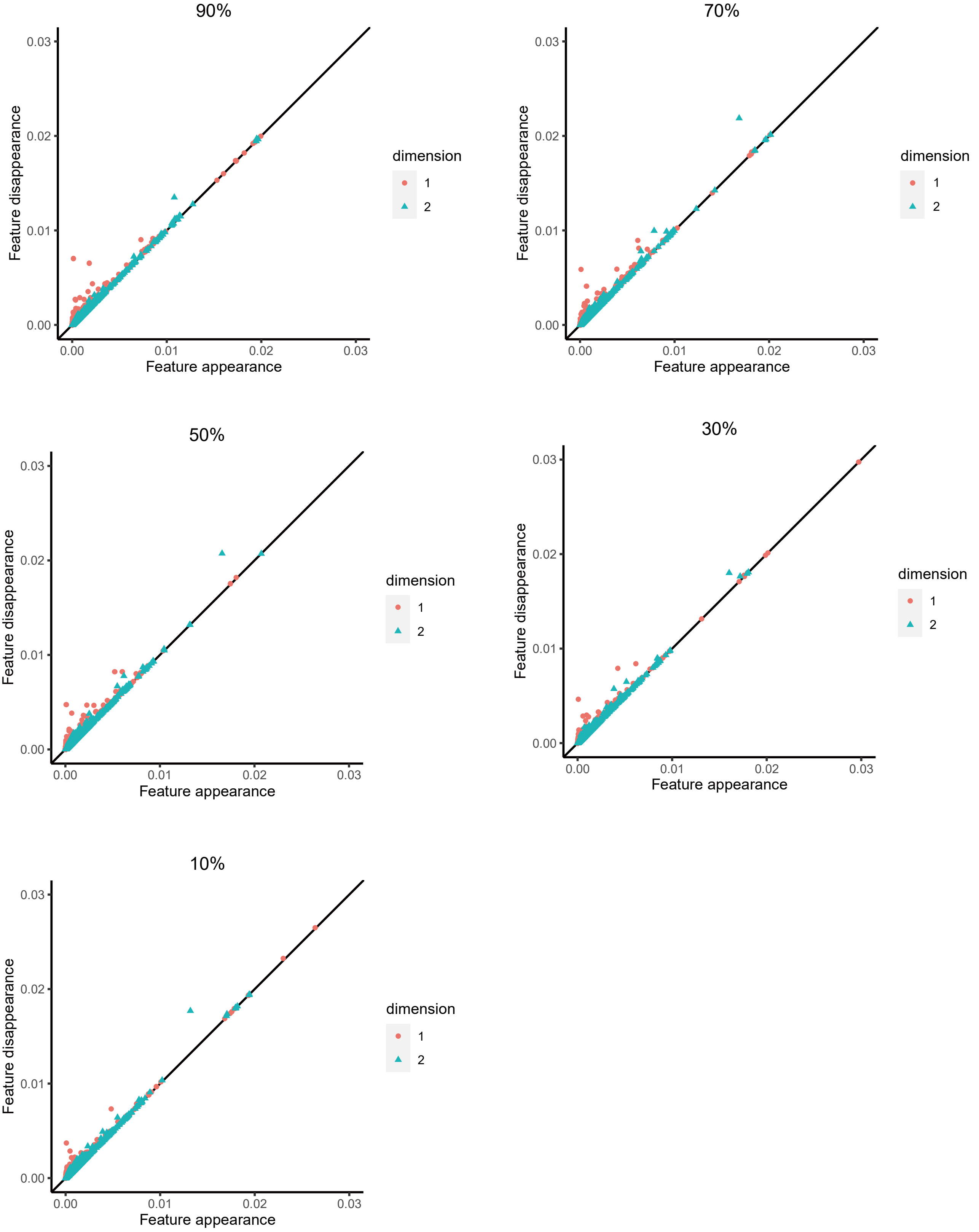

Supplementary Fig. 7 PH analysis with different thresholds of classified signals.

The classified signals with different thresholds of probability (90, 70, 50, 30, 10%) shown in Supplementary Fig. 3 were analyzed with PH. Persistent diagrams (PDs) are shown. The sample is originated from control#1 in Fig. 7. The red points (dimention1) and blue triangles (dimention2) represent planar feature points (loop) and spatial feature points (void), respectively, observed by the persistent homology method.

Supplementary Figure 8

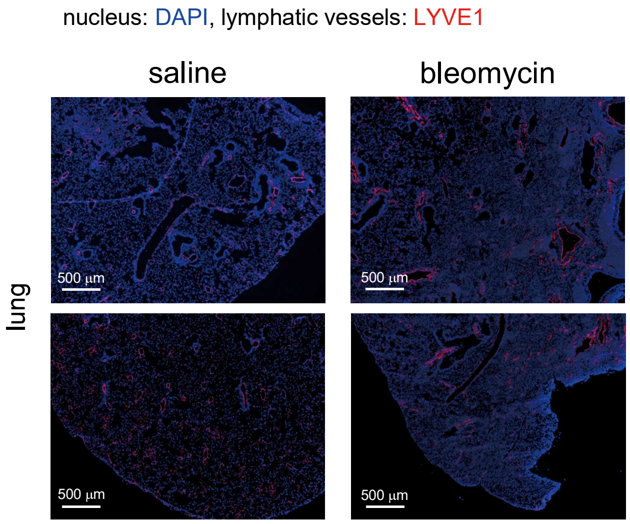

Supplementary Fig. 8 The 2D IHC images of lymphatic vessels in the lung fibrosis model.

Structure of lymphatic vessels determined by 2D IHC. Mice were intratracheally treated with bleomycin to induce lung fibrosis and with saline for control. At 10 to 14 days after instillation, mice were sacrificed, and the lungs were excised. The lung samples were frozen, and the sections were stained with anti-LYVE1 antibody. Representative images are shown.

Supplementary Figure 9

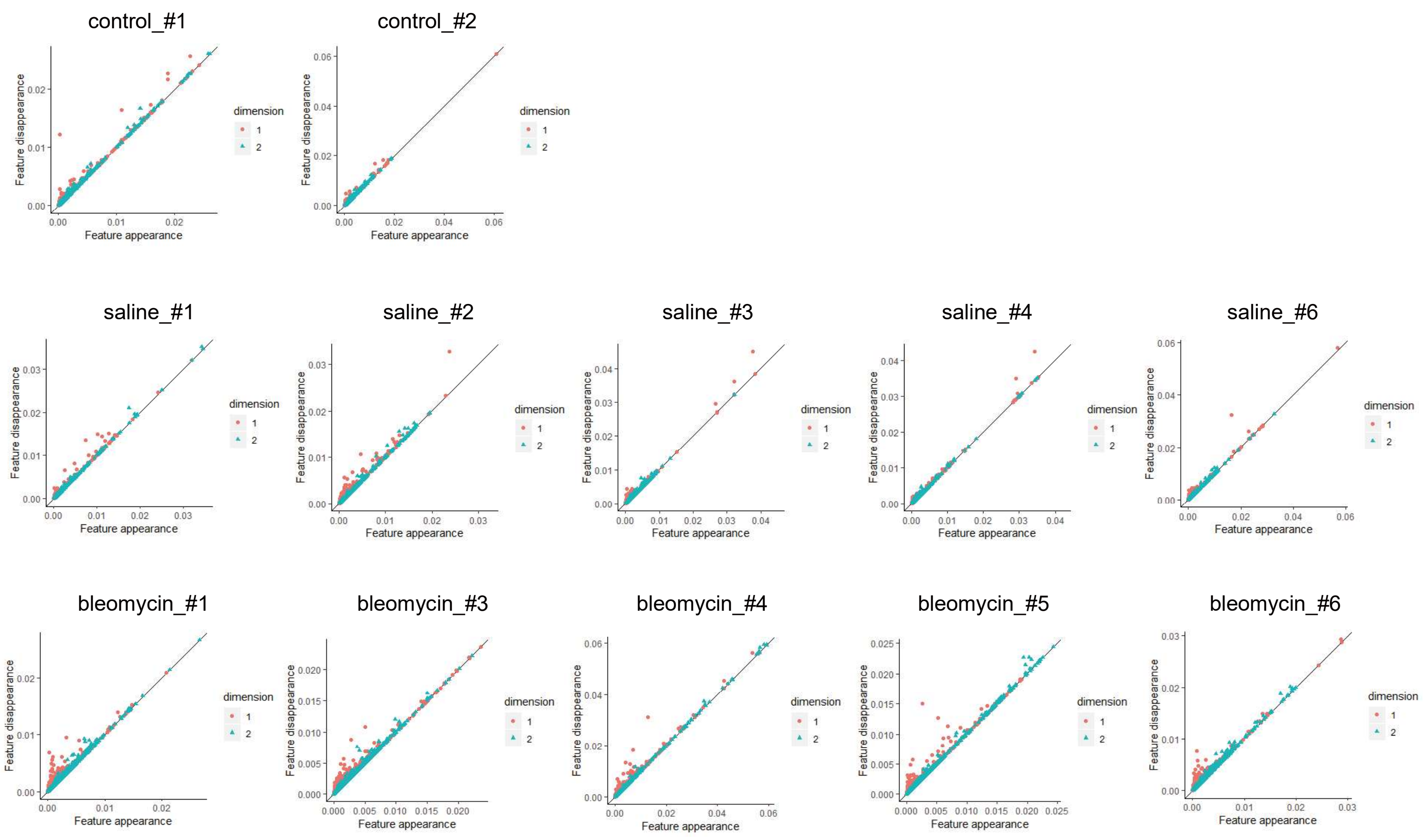

Supplementary Fig. 9 Persistent diagrams of lymphatic vessels in the lung fibrosis model.

Persistent diagrams of mouse lung lymphatic vessels in the fibrosis model as shown in Figure 6. PDs are shown in each sample. The red points (dimention1) and blue triangles (dimention2) represent planar feature points (loop) and spatial feature points (void), respectively, observed by the persistent homology method.

Supplementary Figure 10

a

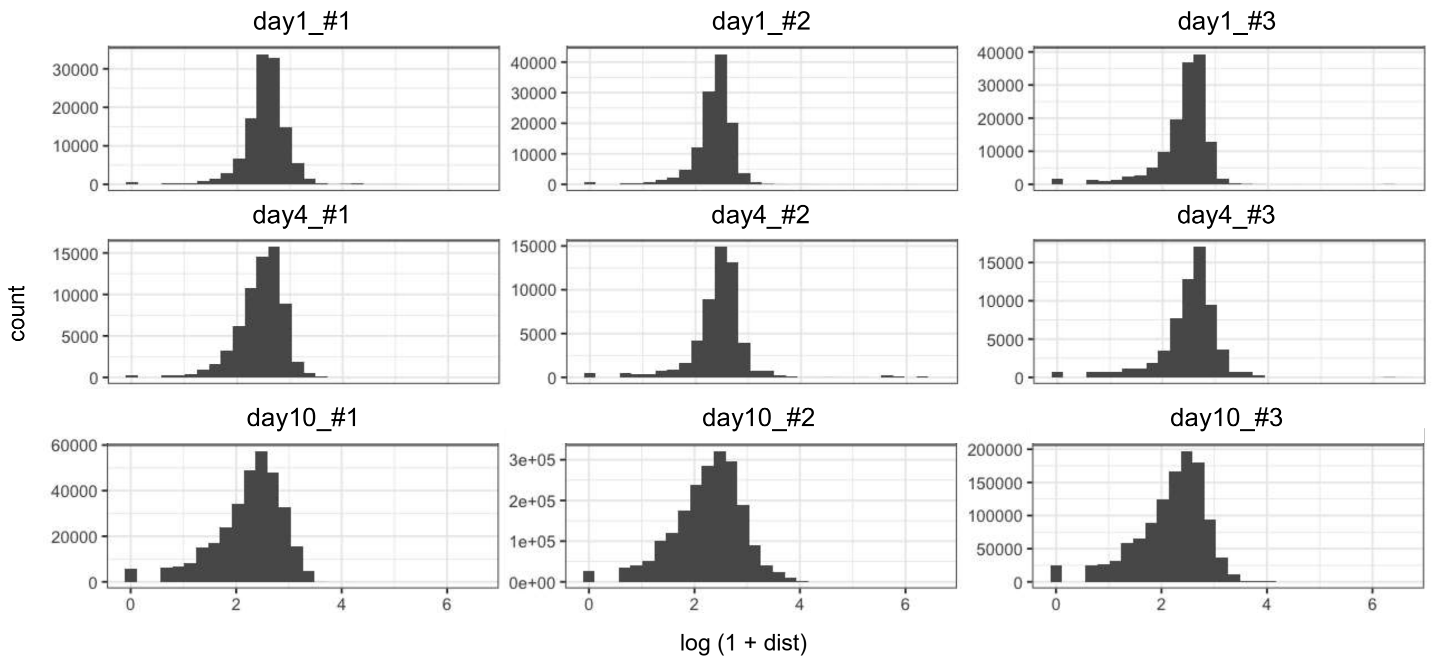

b

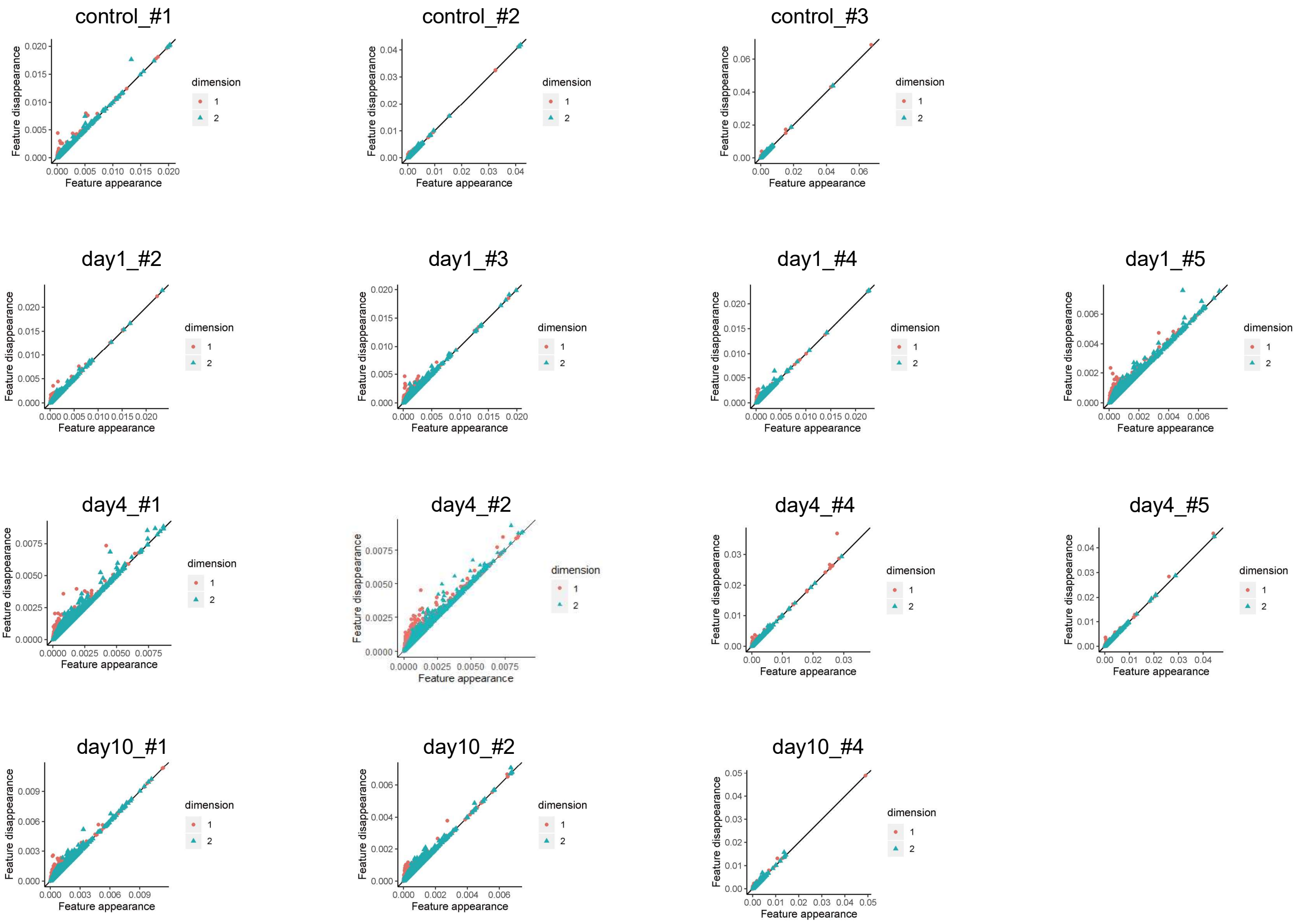

Supplementary Fig. 10 Analysis of lymphatic vessels and mouse melanoma B16F10 lung metastasis.

a The distances between B16F10 cells and lymphatic vessels (pixel-based) are shown in each sample.

b Persistent diagram of the mouse lung lymphatic vessels in B16F10 experimental metastasis model as shown in Figure 7. The red points (dimention1) and blue triangles (dimention2) represent planar feature points (loop) and spatial feature points (void), respectively, observed by the persistent homology method. PDs are shown in each sample.

Supplementary Figure 11

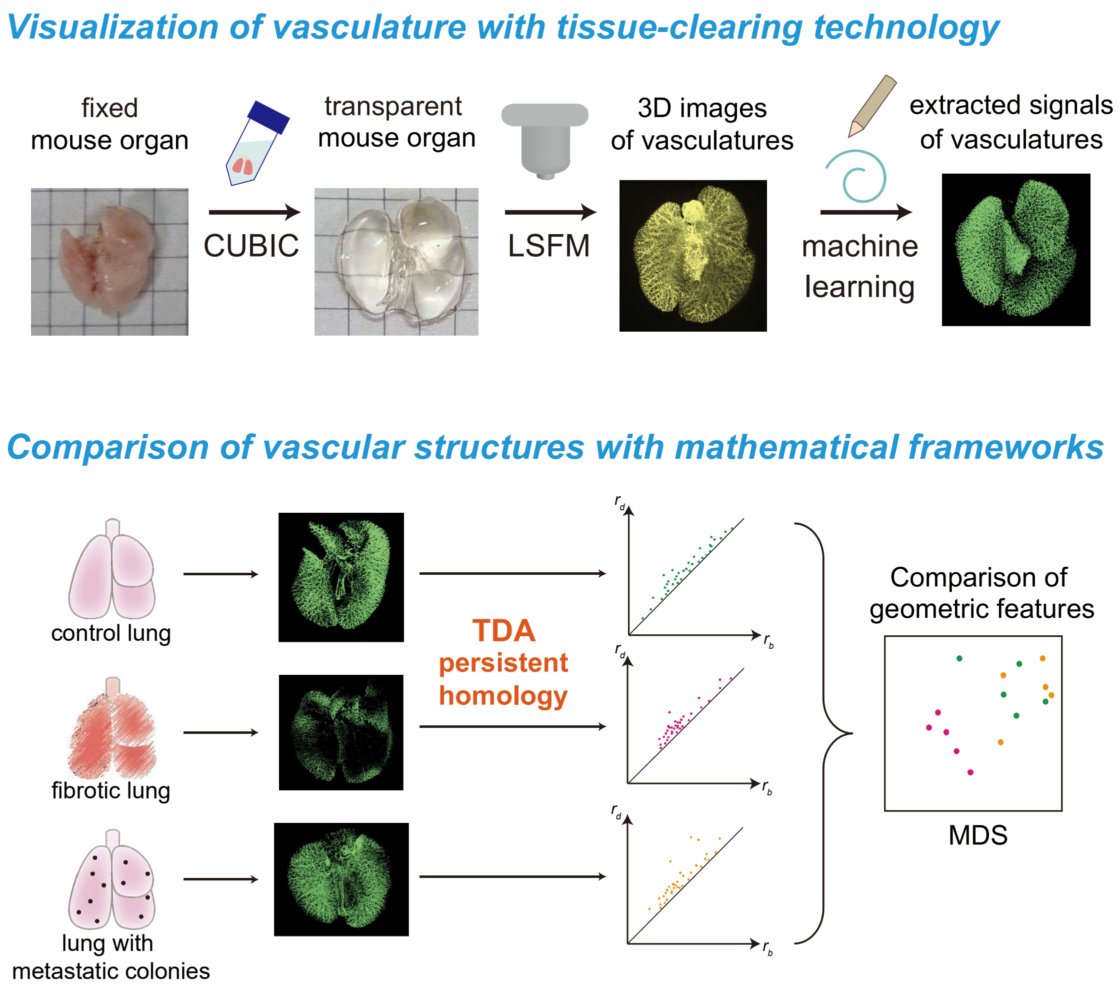

Supplementary Fig. 11 Summary of our current work.
