# Supplementary material for: An analysis modality for vascular structures combining tissue-clearing technology and topological data analysis
Source: Nat Commun. 2022 Sep 12;13:5239. doi: 10.1038/s41467-022-32848-2 (PMC9468184; doi:10.1038/s41467-022-32848-2)
Supplement: Supplementary file 2 — Description of Additional Supplementary Files [file 41467_2022_32848_MOESM2_ESM.pdf]

Title: Supplementary Movie 1.

Description: Visualization of blood vessels in mouse kidney.

Title: Supplementary Movie 2.

Description: Visualization of lymphatic vessels in mouse lung.

Title: Supplementary Movie 3.

Description: Classified signals of brain blood vessels by ilastik.
