# Supplementary material for: An analysis modality for vascular structures combining tissue-clearing technology and topological data analysis
Source: Nat Commun. 2022 Sep 12;13:5239. doi: 10.1038/s41467-022-32848-2 (PMC9468184; doi:10.1038/s41467-022-32848-2)
Supplement: Supplementary file 4 — Reporting Summary [file 41467_2022_32848_MOESM4_ESM.pdf]

nature portfolio

Corresponding author(s): Kohei Miyazono and Teppei Shimamura

Last updated by author(s): Aug 14, 2022

Reporting Summary

Nature Portfolio wishes to improve the reproducibility of the work that we publish. This form provides structure for consistency and transparency in reporting. For further information on Nature Portfolio policies, see our Editorial Policies and the Editorial Policy Checklist.

Statistics

For all statistical analyses, confirm that the following items are present in the figure legend, table legend, main text, or Methods section.

n/a	Confirmed	
☐	☒	The exact sample size (*n*) for each experimental group/condition, given as a discrete number and unit of measurement
☐	☒	A statement on whether measurements were taken from distinct samples or whether the same sample was measured repeatedly
☐	☒	The statistical test(s) used AND whether they are one- or two-sided<br>*Only common tests should be described solely by name; describe more complex techniques in the Methods section.*
☒	☐	A description of all covariates tested
☐	☒	A description of any assumptions or corrections, such as tests of normality and adjustment for multiple comparisons
☐	☒	A full description of the statistical parameters including central tendency (e.g. means) or other basic estimates (e.g. regression coefficient) AND variation (e.g. standard deviation) or associated estimates of uncertainty (e.g. confidence intervals)
☐	☒	For null hypothesis testing, the test statistic (e.g. *F*, *t*, *r*) with confidence intervals, effect sizes, degrees of freedom and *P* value noted<br>*Give P values as exact values whenever suitable.*
☒	☐	For Bayesian analysis, information on the choice of priors and Markov chain Monte Carlo settings
☒	☐	For hierarchical and complex designs, identification of the appropriate level for tests and full reporting of outcomes
☒	☐	Estimates of effect sizes (e.g. Cohen's *d*, Pearson's *r*), indicating how they were calculated

Our web collection on statistics for biologists contains articles on many of the points above.

Software and code

Policy information about availability of computer code

Data collection	In CUBIC whole organ imaging, images were captured using custom-made light sheet fluorescent microscopy (LSFM). In immunohistochemistry, images were captured using AX80 (Olympus) and BZ-X710 microscope (KEYENCE).
Data analysis	The analysis pipeline for this study is available at https://github.com/nagoya-sysbiol/cubic_analysis.<br>ANTs is available at http://stnava.github.io/ANTs/.<br>The python code for TubeMap analysis can be accessed at https://github.com/ChristophKirst/ClearMap2.<br>Persistent diagrams based on persistent homology and the Sliced Wasserstein distances were calculated using HomCloud (version 3.0.1) and the R package kernel TDA (version 1.0.0), respectively.<br>Non-homogeneous Poisson process model was estimated with our original R functions.<br>Fisher's exact test was performed and several plots were generated using R (version 3.6.2).<br>Making graphs and statistical analysis were performed with GraphPad Prism9.<br>Tiff images captured by LSFM were converted using the Imaris File Converter and analyzed by the Imaris software (version 8.4) and Free Imaris Viewer (version 9.5).

For manuscripts utilizing custom algorithms or software that are central to the research but not yet described in published literature, software must be made available to editors and reviewers. We strongly encourage code deposition in a community repository (e.g. GitHub). See the Nature Portfolio guidelines for submitting code & software for further information.

Data

Policy information about availability of data

All manuscripts must include a data availability statement. This statement should provide the following information, where applicable:

- Accession codes, unique identifiers, or web links for publicly available datasets
- A description of any restrictions on data availability
- For clinical datasets or third party data, please ensure that the statement adheres to our policy

Allen Brain Atlas and CUBIC-Atlas are available online (https://portal.brain-map.org/ and http://cubic-atlas.riken.jp/). The data for most figures are provided in the Source Data file. Representative raw image data (tiff files) are available upon request to the authors, due to the data size.

Field-specific reporting

Please select the one below that is the best fit for your research. If you are not sure, read the appropriate sections before making your selection.

[x] Life sciences [ ] Behavioural & social sciences [ ] Ecological, evolutionary & environmental sciences

For a reference copy of the document with all sections, see nature.com/documents/nr-reporting-summary-flat.pdf

Life sciences study design

All studies must disclose on these points even when the disclosure is negative.

Sample size	Sample size for each experiment is indicated in Figure legends. No statical methods were used to predetermine the sample size, however, the sample size were chosen to yield the power to detect the effects.
Data exclusions	No analyses were excluded.
Replication	Each experimnet presented in this study was repeated in multiple times.
Randomization	In vivo experiments, animals were randomly assigned.
Blinding	Blinding of animal studies was not possible due to tagging the animals.

Reporting for specific materials, systems and methods

We require information from authors about some types of materials, experimental systems and methods used in many studies. Here, indicate whether each material, system or method listed is relevant to your study. If you are not sure if a list item applies to your research, read the appropriate section before selecting a response.

Materials & experimental systems

n/a	Involved in the study	
[ ]	[x]	Antibodies
[ ]	[x]	Eukaryotic cell lines
[x]	[ ]	Palaeontology and archaeology
[ ]	[x]	Animals and other organisms
[x]	[ ]	Human research participants
[x]	[ ]	Clinical data
[x]	[ ]	Dual use research of concern

Methods

n/a	Involved in the study	
[x]	[ ]	ChIP-seq
[x]	[ ]	Flow cytometry
[x]	[ ]	MRI-based neuroimaging

Antibodies

Antibodies used	All antibodies used in this study are described in Method section and Supplementary Table4.
Validation	Anti-GFP antibody conjugated with Alexa Fluor 594 (A21312, ThermoFisher) or Alexa Fluor 647 (A31852, ThermoFisher) We can get the reproduced results using either antibody and we use them depending on the filters of LSFM.<br>https://www.thermofisher.com/antibody/product/GFP-Antibody-Polyclonal/A-21312<br>https://www.thermofisher.com/antibody/product/GFP-Antibody-Polyclonal/A-31852<br>Anti-VEGFR3 (AF743, R&D systems)<br>https://www.rndsystems.com/products/mouse-vegfr3-flt-4-antibody_af743<br>Anti-goat conjugated with Alexa Fluor 647 (A21447, Thermo Fisher)<br>https://www.thermofisher.com/antibody/product/Donkey-anti-Goat-IgG-H-L-Cross-Adsorbed-Secondary-Antibody-Polyclonal/

A-21447<br>Anti-LYVE1 (ab14917, abcam)<br>https://www.abcam.com/lyve1-antibody-ab14917.html<br>Anti-CD31 (553370, BD Pharmingen)<br>https://www.bdbiosciences.com/eu/applications/research/stem-cell-research/cancer-research/mouse/purified-rat-anti-mouse-cd31-mec-133/p/553370

Eukaryotic cell lines

Policy information about cell lines

Cell line source(s)	Mouse melanoma cells, B16F10 (American Type Culture Collection), were used in this study.
Authentication	Used cell line was obtained from ATCC and has not been authenticated.
Mycoplasma contamination	We used micoplasma-free B16F10 cells in this study.
Commonly misidentified lines<br>(See ICLAC register)	No commonly misidentified cell lines were used in this study.

Animals and other organisms

Policy information about studies involving animals; ARRIVE guidelines recommended for reporting animal research

Laboratory animals	C57BL/6J mice (5w, female) for lectin injection were purchased from Sankyo Lab Service (Japan). Cdh5-BAC-CreERT2 mice were generated (removed for DBPR) and ROSA-lox-stop-lox-tdTomato mice were obtained from Jackson Laboratory. Prox1-GFP mice were purchased from Mutant Mouse Resource Research Centers (MMRRC). The background of all strains is C57BL/6J. Ages and sex of mice were indicated in figure legend or the Methods section. For vascular visualization (Figure 1, 2 and 3b), Prox1-GFP or VE-cad-tdTomato or VE-cad-tdTomato-Prox1-GFP (male and female, 2-4 months) were used. For brain vascular analysis (Figure 3c, 3d, 4 and 5), VE-cad-tdTomato-Prox1-GFP mice (female, 6-15 months) were used. For lung fibrosis analysis (Figure 6), Prox1-GFP mice (male, 4-16 months) were used. For lung metastasis model (Figure 7), Prox1-GFP mice (female, 3-5 months) were used.
Wild animals	This study did not include wild animals.
Field-collected samples	This study did not include field-collected samples.
Ethics oversight	All experiments were performed under the approval of the Animal Care and the Use of Committee of the Graduate School of Medicine, The University of Tokyo.

Note that full information on the approval of the study protocol must also be provided in the manuscript.
